# "Is Cybermedicine Killing You?" - Peer Review and Evidence-Based Medicine: Author's Reply

**DOI:** 10.2196/jmir.7.4.e39

**Published:** 2005-07-28

**Authors:** Gunther Eysenbach

## Author's Response

Fogel's suggestion of a grading system according to the level of peer review (reminiscent of grading systems for "level of evidence" of primary studies) is interesting, but further study is required to determine to what degree the proposed ratings actually correlate with quality or peer review rigor. My suggestion [1] was to routinely invite all authors of the primary studies to comment on a draft of the systematic review. They actually do not have to peer review the entire paper in the sense of having to write a full referee report, they just should have access to the review before its actual publication to ensure that the authors did not make any major extraction errors (such as in the reported case) or misinterpret any of the original studies (as this would be most easily spotted by the authors of the primary studies). Because authors of systematic reviews often contact the authors of the primary studies anyway (to inquire about nonpublished data or ask other questions), this could be done relatively easily and routinely, in particular, if preprint servers are used, which in other disciplines are common but are underused in medicine.
